# Effects of a novel differential diagnosis aid for managing patients with unexplained fatigue in primary care: a prospective randomized, controlled, open and multicenter study in primary care

**DOI:** 10.1186/s12875-025-02873-3

**Published:** 2025-05-24

**Authors:** Roland von Känel, Stefan Neuner-Jehle, Reto W. Kressig, Idris Guessous, Pierre Alexandre Krayenbühl, Lukas Zimmerli, Anne Angelilo-Scherer, Thomas Keller, Caroline Elzner, Karl Pauls, Neige Morin, Edouard Battegay

**Affiliations:** 1 Department of Consultation-Liaison Psychiatry and Psychosomatic Medicine, University Hospital Zurich, University of Zurich, Zurich, Switzerland; 2 Institute of Primary Care, University of Zurich and University Hospital of Zurich, Zurich, Switzerland; 3 University Department of Geriatric Medicine Felix Platter &, University of Basel, Basel, Switzerland; 4 Unit of Population Epidemiology, Department of Community Medicine, Primary Care and Emergency Medicine, Geneva University Hospitals, Geneva, Switzerland; 5 General Practice, Brauereistrasse, Uster, Switzerland; 6 Department of Internal Medicine, Cantonal Hospital Olten, Solothurner Spitäler AG, Olten, Switzerland; 7 Department of Haematology and Central Haematology Laboratory, University Insel Hospital, Bern, and Medical Faculty, Basel University, Basel, Switzerland; 8 ACOMED Statistik, Leipzig, Germany; 9 Berlin Center for Epidemiology and Health Research, ZEG, Berlin, Germany; 10 Medical Affairs Department, CSL Vifor, Zurich, Switzerland; 11 International Center for Multimorbidity and Complexity in Medicine (ICMC), UZH Longevity Center, University of Zurich, Merian Iselin Klinik, Basel, Switzerland

**Keywords:** Fatigue, Differential diagnosis, Treatment outcome, Patient relevant outcome, Patient satisfaction, Primary care, Clinical trial

## Abstract

**Aims of the study:**

Unexplained fatigue is a common reason for encounters in primary care. However, currently no aid orients physicians in detecting its potential causes. The aim of this study was to evaluate whether the novel Fatigue Differential Diagnostic Aid (FDDA) supported clinicians in better managing unexplained fatigue.

**Methods:**

This was a prospective, cluster-randomized, controlled, open, and multicenter study comparing the use of the FDDA vs usual care in patients with unexplained fatigue as the main reason for encounter. The primary endpoint was difference in Patient Global Impression of Change (PGIC) between groups at 3 months. Among pre-defined secondary endpoints were: Difference in change of PGIC between groups at 6 months; percentage of patients with fatigue reduction; mean reduction in fatigue; clinician’s confidence in diagnosis; patient satisfaction with quality of care (diagnostic process and treatment); number of clinician-reported visits; number of referrals to specialists; and time until final diagnosis.

**Results:**

112 primary care practitioners (PCPs) recruited in Switzerland between 2017 to 2020 were randomly cluster-assigned to the FDDA = 57 or usual care = 55 arm. Of these, 15 (FDDA) and 22 (usual care) PCPs recruited 93 patients (FDDA: *n* = 40, usual care: *n* = 53). The achieved sample size was less than planned. There was no difference in PGIC at 3 months between groups (D = 0.06, 95%-CI: -0.41 – -0.53, *p* = 0.802). Among secondary endpoints, no significant differences occurred in PGIC at 6 months, nor in fatigue reduction. However, in the FDDA group, more patients reported less fatigue at 3 or 6 months (D = 18.9%, 95%-CI: -33.6 – -4.3%, *p* = 0.011), and increased satisfaction with treatment management at 1 month (FDDA 56.8% vs usual care 25.0%, *p* = 0.004) and 3 months (FDDA 64.9% vs usual care 31.0%, *p* = 0.003); the FDDA was also associated with higher total number of visits (median 4.0 vs 3.0, *p* < 0.001).

**Conclusions:**

In this pilot study, the FDDA, a structured diagnostic aid for guiding PCPs in identifying the causes of unexplained fatigue in their patients, was not able to show a global improvement in patient outcomes despite improvements in fatigue and satisfaction with care. The evaluation of fatigue in larger-scale studies is warranted.

**Trial registration:**

This trial was retrospectively registered on ClinicalTrials.gov. Trial registration number: NCT05861492. Date of registration: 17th May 2023. The ethics committee of Ethikkommission Nordwest- und Zentralschweiz (EKNZ) had originally voiced the opinion that no registration was required because no drug or intervention was involved, i.e., the study was non-interventional and observational. However, the study authors felt that the study should be retrospectively registered because the FDDA could be interpreted to be an active intervention. At the time of registration, two protocol deviations occurred that are explicitly addressed in the Methods section of this manuscript. Because of the low sample size, we statistically compared “patients” instead of “comparing patients nested in doctors” (the latter was performed as an additional analysis). Thus, cluster randomization was performed, but the analysis to consider this was not feasible.

**Supplementary Information:**

The online version contains supplementary material available at 10.1186/s12875-025-02873-3.

## Introduction

Fatigue is highly prevalent in the general population, with up to 15% of patients presenting with fatigue and 27% of primary care physicians (PCP) reporting this as a frequent patient complaint in primary care [1–4]. In up to 8% of patients, fatigue is reported as the main reason for encounter [5]. Fatigue can significantly impact a person’s well-being and performance and then become clinically relevant.

**Fig. 1:**
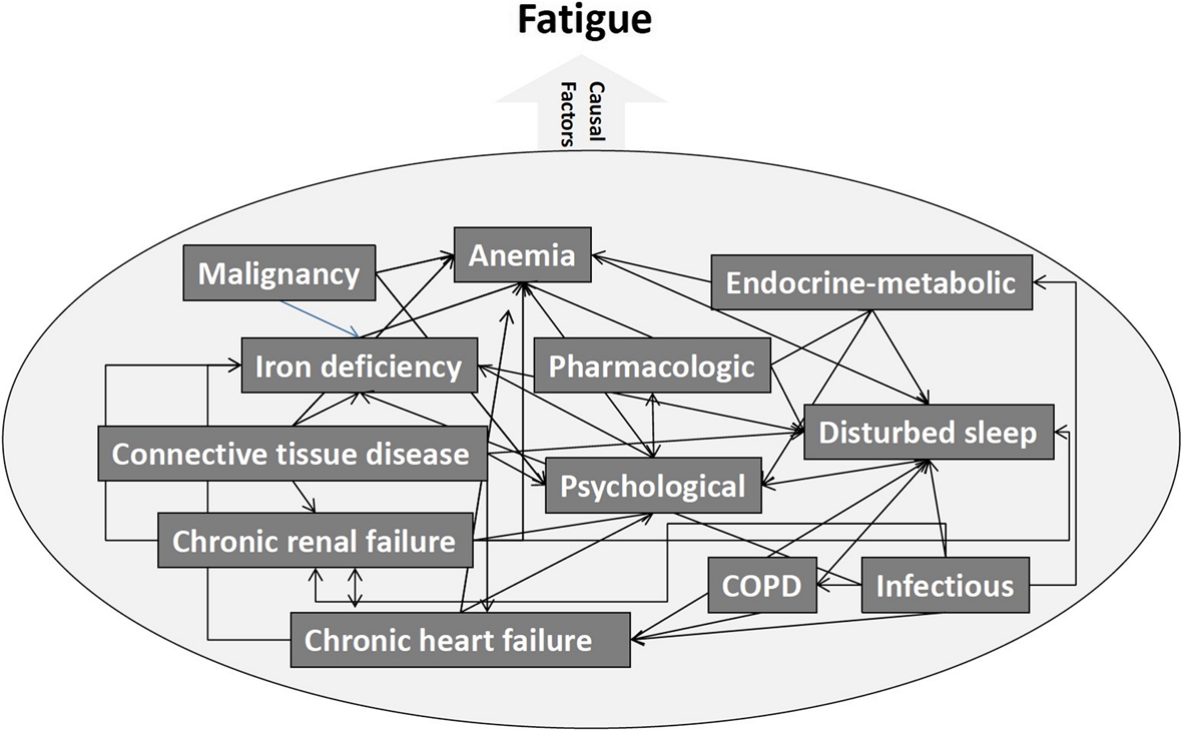
Underlying and potentially interacting causes of fatigue

When describing their fatigue, patients use terms such as generalized weakness, inability to initiate certain activities, being easily tired, reduced capacity to maintain performance, impaired concentration or mental disturbances, sometimes associated with depression and anxiety, [6] to tiredness, exhaustion, lack of energy, sleep disorders, dizziness, or concentration problems [7]. Concepts to assess and deal with these complaints may diverge between patients and their treating physicians. Thus, PCPs often detract from the leading symptom and reason of encounter of “fatigue” because of the patient’s concealed presentation. The latter may also camouflage diagnoses associated with the core symptom of fatigue. Thus, description of fatigue is often non-specific, difficult to classify, and associates with a wide range of differential diagnoses [8–10]. Yet, a clear and specific diagnosis is necessary because fatigue can indicate potentially life-threatening underlying diseases or conditions of organic, [11–13] mental, behavioral, [7, 14–16] or lifestyle [7, 17] origin. Interestingly, the etiology of fatigue may remain unclear in up to one-third of patients [18]. The work-up for the differential diagnosis of fatigue is complex, often time-consuming, and unsatisfactory for patients, physicians and their interactions [19, 20]. For a brief overview of the complexity of potential underlying and interacting causes of fatigue, see Fig. 1.

A rational and structured approach supports attaining a specific diagnosis of the underlying causes of fatigue. Differential diagnostic aids for fatigue have been developed for already diagnosed health conditions, such as pain, cancer, [21–24] arthritis, [25] or multiple sclerosis [26]. Also, several tools based on patient-reported outcomes help assess the intensity and impact of fatigue, including the Epworth Sleepiness Scale that evaluates daytime sleepiness, the STOP-Bang questionnaire for obstructive sleep apnea, or the Patient Health Questionnaire-9 (PHQ9) that monitors depression [9]. Reviews summarize the differential diagnosis of unexplained fatigue, [17, 27] but no tool appears to support PCPs in the differential diagnosis of unexplained fatigue. To fill this gap, the multidisciplinary Swiss Fatigue Working Group (SFWG), composed of physicians selected based on their published work in fields relevant to fatigue, focused on developing the Fatigue Differential Diagnostic Aid (FDDA)(see Appendix 1, Supplementary material). The FDDA was designed to guide the clinician towards potential causes of unexplained fatigue of organic, mental, behavioural, or lifestyle origin to enable a more structured, evidence-based, and efficient course of investigations and treatment decisions to be made.

The FDDA includes validated questions from well-published tests, such as the PHQ-2, PHQ-5 and GAD-7, and other clinically relevant questions to assess fatigue. Briefly, in a preliminary feasibility phase of this study (data not shown), participating PCPs assessed patients with unexplained fatigue using an initial version of the FDDA. Patients were asked to provide a detailed account of their condition, its impact on their lives, and insights on how fatigue was addressed during doctor encounters. Based on the information gathered, the FDDA was amended and submitted to the patients for qualitative feedback, and to the physicians for content, relevance, accuracy, and feasibility. It was estimated that physicians would need 20 min to complete the FDDA when using it for the first time with a patient. During the feasibility phase, both PCPs and patients considered this time appropriate for investigating unexplained fatigue. The SFWG supervised every step of the development of the FDDA, oversaw the project throughout its duration and made the final adaptations to the aid for use in this study. The aim of this study was to evaluate how the novel Fatigue Differential Diagnostic Aid (FDDA) supported and improved clinical management and care of patients with unexplained fatigue in primary care.

## Methods

### Study design

This was a prospective, randomized, controlled, open and multicenter study involving neither treatment intervention nor study drug, conducted in primary care practices across Switzerland. The study was planned and conducted in 2 Phases. Phase 1 was planned to collect data at baseline and reflect on current practice and is not described in this study. Phase 2 was designed to compare the use of FDDA vs usual care.

### Primary care physician recruitment

PCPs were recruited between 2017 and 2020 from all Cantons of the Swiss-German and the Swiss-French speaking parts of Switzerland by a Field Organization (Qualipro). The study was cut short when the Covid 19 pandemic started, and patient recruitment had to stop.

The study questionnaires were provided in the local language. PCPs specialized in general or internal medicine who saw up to 5 patients per week with unexplained fatigue as the main reason for encounter were recruited. Exclusion criteria were: Working in, or affiliated with, an “iron center” (a medical center known to be primarily inclined to prescribing intravenous iron supplementation in cases of fatigue); known to be experienced in fatigue or chronic fatigue syndrome; seeing more than 5 patients per week for fatigue; specialized (i.e., certified continuing education) in psychosomatic medicine.

### Randomization

PCPs who met the inclusion criteria were consecutively assigned on a 1:1 ratio by cluster randomization either to the FDDA or the usual care study group at baseline, using a fixed (SAS code with a fixed seed [28] block-size of 8 and based on the time point of documentation of recruitment in the study database. A random allocation sequence list was used. To prevent bias, PCPs randomized to usual care were not informed about the existence of FDDA.

### Patient recruitment

PCPs recruited their patients. Patients were recruited by the PCP and were regarded to be nested within a PCP (PCP = cluster). Recruitment was set at 3 patients per PCP. However, some PCPs were unable to reach this target. To counterbalance this, a protocol amendment was made to increase the number of patients per PCP to 4.

Patient inclusion criteria were adults aged between 18 to 80 years with fatigue of unexplained origin as the main reason for encounter; symptoms of fatigue lasting for at least 2 weeks but no longer than 2 years before inclusion. Patient exclusion criteria were: pre-existing anaemia; treatment received for fatigue by a physician within 3 months prior to the baseline visit; previously diagnosed disease that could have induced the fatigue symptoms (e.g. chronic heart or kidney failure; inflammatory bowel disease; rheumatoid arthritis, multiple sclerosis, cancer); known use of medications could induce fatigue-like symptoms, such as antihistamines, antidepressants, benzodiazepines, hypnotics, anxiolytics, opioid formulations (this list is non-exhaustive and medical judgement could be applied); participating in, or having completed another clinical trial < 30 days prior to study entry. All patients who participated in the study provided informed consent. Patient data was handled in accordance with the European data privacy regulation.

### Study procedures

**Fig. 2:**
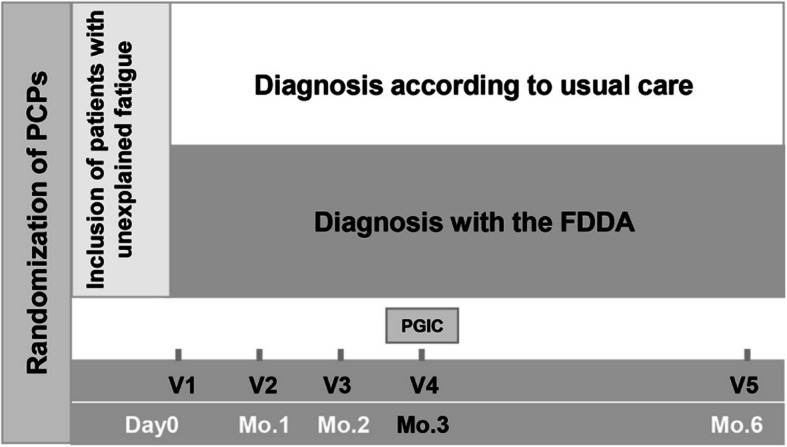
Study design. Recruited PCPs were cluster randomized either into the FDDA or usual care study arm. Each physician recruited up to 3 patients with unexplained fatigue as the main reason for encounter. Diagnosis was made using either the FDDA (FDDA arm) or following usual care practice. The primary endpoint was difference in PGIC between FDDA vs usual care at 3 months (planned visit 4). FDDA, Fatigue Differential Diagnostic Aid; V, visit; Mo, month; PCP, primary care practitioner; PGIC, Patient global impression of change questionnaire

PCPs in group 1 (usual care) continued to manage their patients with unexplained fatigue as usual, whereas group 2 (FDDA) PCPs integrated the FDDA in the diagnostic process. Patients were assessed at baseline (visit 1), and then at 1 month (visit 2), 2 months (telephone consultation, visit 3), 3 months (visit 4) and 6 months (telephone consultation, visit 5) (see Fig. 2). The baseline, 1- and 3-month visits were performed by the treating physician, and the 2- and 6-month follow-up visits were carried out by the field organization as telephone consultations.

### Study endpoints

The primary endpoint was difference in Patient Global Impression of Change (PGIC) between the FDDA vs usual care groups at 3 months. A clinically significant response was defined as a difference in PGIC of ≥ 1 point, and the FDDA intervention would be regarded as successful if the proportion of positive PGIC responders was ≥ 15% higher compared to usual care. The PGIC questionnaire is a 7-point scale that assesses a patient's perceived impact of disease management, with a 1-point or greater change compared to baseline indicating a clinically meaningful improvement/deterioration (1 = “very much improved”, 7 = “very much worsened”). The PGIC questionnaire was completed by patients at 1, 2, 3, and 6 months.

A detailed list of predefined secondary endpoints is provided in the supplementary material (Supplementary Table 1). Briefly, the FDDA group was compared with the usual care group for (2) PGIC at 6 months; (3) percentage of patients experiencing fatigue reduction ≥ 1 point on a 1–10 numerical rating scale (NRS) at 3 or 6 months vs baseline, (4) time until improvement of fatigue ≥ 1 point; (5) mean number of points of fatigue reduction on a 1–10 NRS scale vs baseline; (6) percentage of patients with a PGIC response (any improvement vs no improvement) at 3, 6, and 3 or 6 months; (7) PCP confidence in diagnosis at 1 and 3 months; (8) Clinical Global Impression of Change (CGIC) at 1 and 3 months; (9a) patient satisfaction with the quality of care related to the diagnostic process at 1, 2, 3 and 6 months; (9b) patient satisfaction with treatment at 1, 2, 3 and 6 months; (10) number of PCP-reported visits (there was fixed number of mandatory onsite visits for outcomes assessment, including visit at baseline, 1 month and 3 months, however, more visits were allowed if judged necessary by the PCP); (11) number of any specialist referrals (imaging or other health services) required by the PCP for a more accurate diagnosis; (12) time until final diagnosis, evaluated at 1 and 3 months.

Further exploratory endpoints were assessed and analyzed: number of physician-reported investigations at baseline, including laboratory investigations, imaging, and psychological evaluations; type of diagnosis for fatigue at baseline (the diagnoses provided by the PCPs were coded by the CRO using the International Classification of Diseases-10, ICD-10); and variety of treatments prescribed for fatigue reported by PCPs for each patient at 1 and 3 months.

### Statistical analyses

The planned sample size (144 PCP and 432 patients) was calculated based on paired data for PGIC, whereby the crosstabulation was simplified to a 2 × 2 table. Assumed a 15% between-arm difference in binary response, the sample size for McNemar-Test was determined (alpha-level 0.05, power 80%). Considering a design effect factor of 1.3 and 15% dropouts, the final sample size of 144 GP was yielded. This sample size (72 doctors per study arm) was also considered to be appropriate to perform logistic regression considering 4 factor withing matching procedures.

Comparisons were planned with an alpha level of 0.05 and 95% confidence intervals. A complete case analysis was planned. Missing values would not be imputed. No correction of the alpha-level in terms of multiple testing of the secondary endpoint was foreseen.

Descriptive statistics were presented for continuously scaled variables (mean, standard deviation, median, Q1, Q3, min, max) and categorically scaled variables (counts and percentages).

The analysis sets were defined as follows: Full analysis set (FAS): all patients intended for application of the FDDA, with at least one visit and available study data. All analyses refer to this population. Per protocol set (PPS): all FAS patients with no major protocol violations, i.e., fulfilling inclusion/exclusion criteria with available data for primary variable (PGIC at 3 months). The main analyses were repeated for this population (results not shown).

For the analysis of the group comparisons a methodology according to the clustered structure was planned at PCP level with nested patients (mixed models). Moreover, matched analysis of the PCPs was planned considering 4 factors: size of practice, medical specialty, years of experience, as well as region (town, village, and dominant language). Propensity score matching was foreseen.

Since the achieved sample size was considerably lower than the planned samples size (planned: 144 PCPs, 432 patients, realized: 112 PCPs, of which 37 recruited 93 patients), the SAP was used to adapt the analysis. Since appr. 1/3 of the PCPs recruited only 1 patient, the mixed model is difficult to handle. Moreover, regression modelling which applies 4 predefined factors for matching was not possible anymore. Instead, univariable between-group (FDDA vs usual care) comparisons were conducted at patient level. Endpoints (1), (2), (5), (7) and (8) were analyzed by two-sided two-sample *t*-test. Endpoints (3), (6), (9a) and (9b) were analyzed by Fisher’s exact test. For the primary and secondary endpoints (2), (5), (7), (8) and (9), sensitivity analyses were performed that accounted for the data's clustered structure by using mixed models with PCP as a random factor (results are reported in Supplementary Table 2). Endpoints (4), (10), and (12) were analyzed by Mann–Whitney-U-Test. Additionally, for (10) the number of required visits were analyzed as categorically scaled variable by Mantel–Haenszel Chi-square test. The differences between FDDA and usual care groups regarding all treatment options (“no treatment”, “only iron replacement therapy”, “iron replacement therapy combined with other treatments”, or “only other non-iron treatments”) were statistically investigated by a 4 × 2 contingency table. The Shannon’s H-Index was used to compare variety of treatments (no treatment, only iron replacement therapy, iron replacement therapy combined to other treatments, or only other non-iron replacement treatments) between the FDDA and usual care groups.

The methodology applied for reporting results follows the CONSORT guidelines and checklist [29] (Supplementary File 3).

## Results

**Fig. 3:**
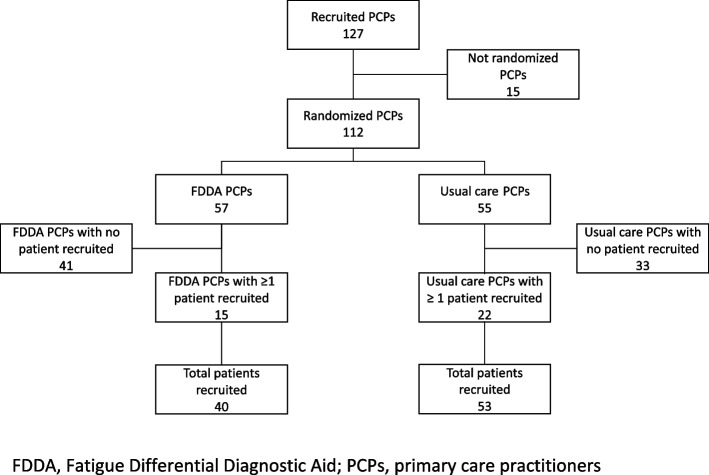
Study flowchart. FDDA, Fatigue Differential Diagnostic Aid; PCPs, primary care practitioners

A total of 112 PCPs included between 2017 and 2020 were randomly assigned either to the FDDA or the usual care group (FDDA = 57, usual care = 55). Out of these, 15 (FDDA) and 22 PCPs (usual care) recruited at least 1 patient. A total of 93 patients were recruited (FDDA = 40, usual care = 53). Because the PCPs experienced continued difficulties in recruiting patients, the study was then stopped. Details are given in the flow chart, see Fig. 3.

Primary care practitioner characteristics at baseline included age, gender and level of experience, and were comparable between the two groups. All physicians specialized in general and/or internal medicine, see Supplementary Table 3. Patient characteristics at baseline were comparable in both groups and are summarized in Table 1.

**Table 1:** Patient characteristics at baseline

	**FDDA (*n* = 40)**	**Usual Care (*n* = 53)**
Mean age, years (range)	39.6(18–77)	37.2(17^#^−78)
Sex, female, %	80.0	81.1
Intensity of fatigue at baseline^a^ mean (range)	7.0 (3–9)	6.9 (3–10)

^a^Intensity of fatigue at baseline was measured on a scale of 0 (”no fatigue”) to 10 (“extreme fatigue”). # Patient with protocol violation (17y) was included in the FAS

The most frequently reported diagnosis that PCPs made to explain fatigue at baseline was iron deficiency (44.4% of all diagnoses). Other diagnoses included deficiency in vitamin D or vitamin B, post-viral fatigue syndrome, problems related to employment/unemployment, problems related to life-management difficulties, somatoform disorders, sleep disorders and chronic sinusitis.

The primary endpoint was not met for difference in PGIC at 3 months (FDDA 2.70 vs usual care 2.64; ∆ = 0.06, 95%-CI: −0.41 – 0.53, *p* = 0.802). Results for all other endpoints, including secondary endpoints that did not reach statistical significance, are available in Supplementary Table 1. Regarding fatigue, the percentage of patients who experienced a reduction of ≥ 1 point after 3 or 6 months was significantly higher in the FDDA group compared with usual care (97.4% (95%CI: 90.96% – 100.00%) vs 78.4% (95%CI: 66.16–90.70); *p* = 0.011). All other fatigue-related endpoints did not show significant improvement.

**Fig. 4:**
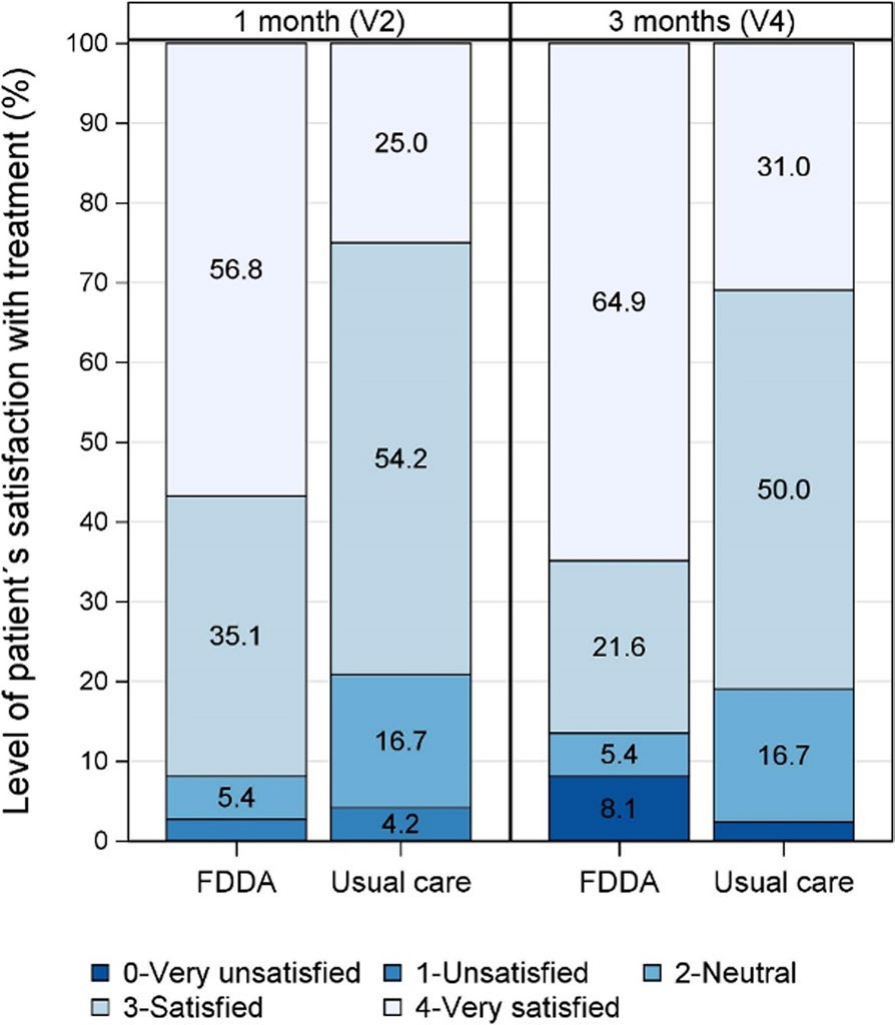
Level of patient satisfaction with treatment at 1 and 3 months. FDDA, Fatigue Differential Diagnostic Aid; V, visit

Regarding the targeting and managing treatment, several secondary endpoints were statistically and clinically significant. When compared with usual care, more patients evaluated with the FDDA were “very satisfied” with the treatment they received at 1 and 3 months (56.8% vs 25.0%, *p* = 0.004 and 64.9% vs 31.0%, *p* = 0.003, respectively), Fig. 4.

**Fig. 5:**
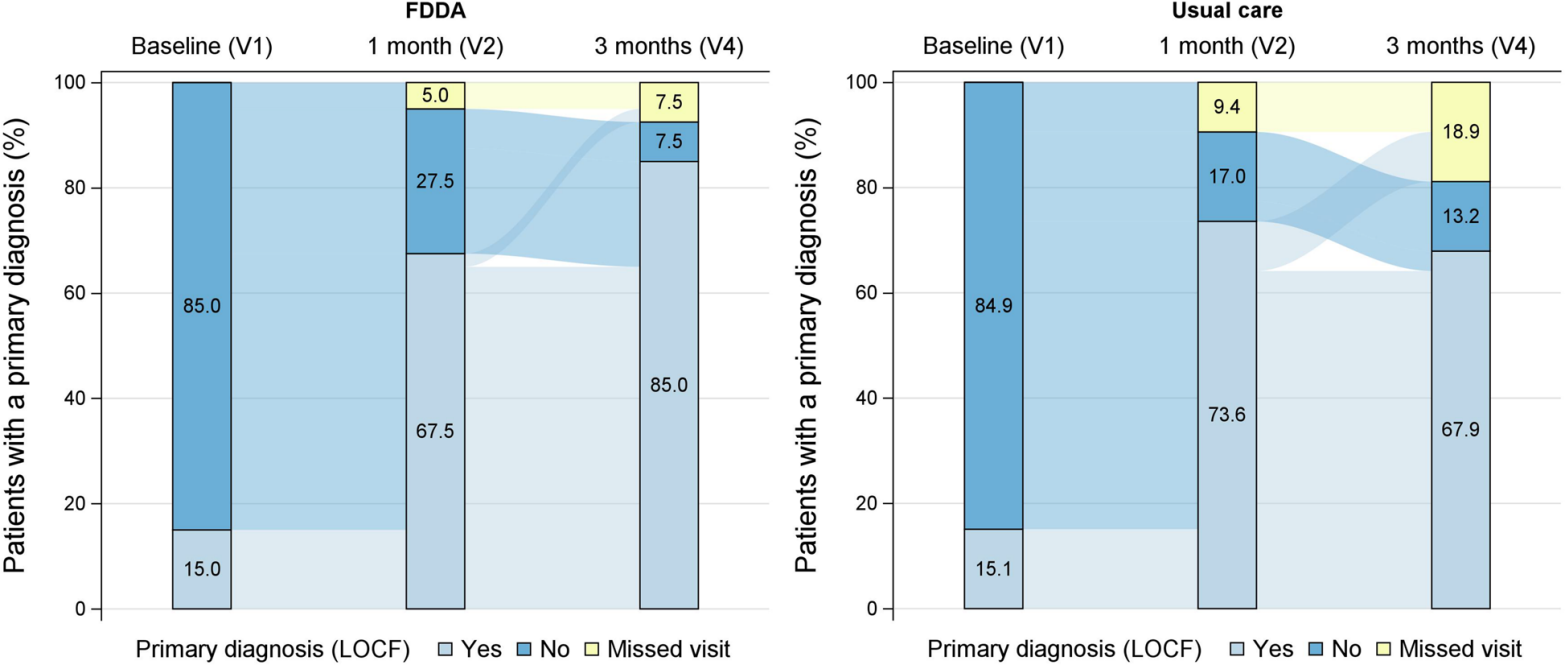
Proportion of patients who received a primary diagnosis for the cause of fatigue. For the primary diagnosis, the last observation was carried forward in case of missing values, but not if values were missing due to missing visits. FDDA, Fatigue Differential Diagnostic Aid; LOCF, last observation carried forward

In the FDDA group, at visit 4 less patients had dropped out (7.5% vs. 18.9%, n.s.), more patients had received a primary diagnosis for the cause of fatigue (85.0% vs. 67.9%, Fisher’s exact test: *p* = 0.088), fewer patients were left untreated, and fewer patients had received iron supplementation only, as illustrated in the alluvial plots in Fig. 5 (see also Supplementary Fig. 1).

Differences in treatments delivered to the two study groups were observed at 1 month (*p* = 0.0233). Less patients in the FDDA group remained without treatment (FDDA 21.1% vs usual care 33.3%) or received iron replacement therapy only (FDDA 21.1% vs usual care 39.6%) (see Supplementary Fig. 1). However, the FDDA group received more other treatments combined with iron (18.4% vs 4.2%) or other treatments only (39.5% vs 22.9%) compared to usual care. At 3 months, the same pattern was observed, but less pronounced (*p* = 0.46). In the FDDA group, a numerically greater variety in treatments was observed at 1 month [FDDA 2.03 (95%CI: 1.74–2.31) vs usual care 1.55 (95%CI: 1.25–1.86), *p* = 0.764] and at 3 months [FDDA 2.26 (95%CI: 1.88–2.63) vs usual care 1.77 (95%CI: 1.45–2.10), *p* = 0.793]. See Supplementary Figs. 2 and 3 for treatments delivered.

The median number of visits per patient to the PCP’s practice for unexplained fatigue was 4 in the FDDA group vs 3 in the usual care group (*p* = < 0.001), demonstrating a strong difference between groups.

Finally, for the exploratory endpoint, the median number of investigations reported by physicians at baseline was higher in the FDDA vs usual care group (median 11.0 (quartile Q1: 8, Q3: 17) vs 7 (Q1: 3, Q3: 11), *p* = 0.002).

## Discussion

Currently, there is no gold standard or accepted systematic approach to support PCPs in differentiating etiologies of fatigue in the general practice setting. To our knowledge, this is the first study to evaluate use of a differential diagnostic aid to facilitate PCPs’ task in addressing a patient’s cause of unexplained fatigue.

In our study, PGIC improved in both groups, however, the difference in PGIC between the two groups at 3 months did not reach significance, and thus the primary endpoint was not attained.

Among secondary endpoints, use of the FDDA was associated with a higher level of patient satisfaction with treatment received compared with usual care. This may be the result of a more intensive diagnostic workup and increased care associated with the FDDA. This observation is supported by our other significant finding, i.e., a higher number of physician-reported investigations and visits in the FDDA group. Thus, a structured diagnostic aid can impact patient-physician interactions, including the number of encounters and extend of diagnostic procedures. Furthermore, a higher percentage of patients in the FDDA group reported reduced fatigue and fewer patients were left untreated in the FDDA group, although iron replacement was prescribed less frequently. Thus, more non-iron treatments were prescribed to patients in the FDDA group, which may be due to the FDDA prompting a more in-depth investigation into the causes of patient reported fatigue. In effect, PCPs in the FDDA group spent more time investigating these patients, which in turn may have given patients a sense of empowerment and strengthened their adherence to investigations and treatments, as well as to behavioural changes suggested by their treating physician. In summary, the use of a diagnostic aid may have helped PCPs to better manage patients with unexplained fatigue. However, this remains speculative and would require further research [30, 31]. Overall, our results highlight the clinical value of providing PCPs with a systematized, comprehensive aid for improved management of patients with unexplained fatigue.

While this study has several strengths, several limitations that may be inherent to non-treatment intervention studies carried out in a real-world setting need to be further discussed. The smaller than planned number of patients recruited and the higher-than-expected dropout rate of patients at follow-up is a significant limitation. This occurred despite extending the recruitment phase of the study and the measures taken to increase the number of participating physicians. As a result, the planned matched analyses could not be performed, and a simple comparison was used for primary analysis instead of mixed models. The power of some endpoints was compromised, leaving several questions unanswered. The difficulties hampering recruitment may be indicative of the complexity of delineating, diagnosing, treating, and managing patients presenting with unexplained fatigue or corresponding synonyms. Additionally, the outbreak of the COVID-19 pandemic, especially the first wave when PCPs were overwhelmed with work, may have heavily impacted PCPs’ capacity to contribute to research projects [32–34]. The overall dwindling of recruitment rates despite measures taken led to the decision to stop the study. Another limitation of the study could be the overestimation of the number of patients presenting with unexplained fatigue. However, these estimates were based on reports from physicians in the feasibility study, which appeared to align with findings from the literature. Despite this, relevant secondary endpoints reached both statistical and clinical significance, suggesting that the impact of the FDDA was greater than anticipated by the SFWG. This appears to have been the case even though the participating physicians were aware of the aim of the study, which could have led them to automatically pay more attention to the diagnostic process in the standard care group, thereby reducing the effect size of endpoints. A further limitation may have been that PCPs selected patients with specific extent or types of fatigue to be included into the study. Yet, the levels of fatigue reported for patients at baseline were almost identical across groups, which speaks against a bias in the conclusions reached. The study was planned to avoid information bias by not informing PCPs and patients of the existence of the other arm using cluster randomization. However, cross-awareness cannot be fully excluded. Also, a placebo effect can be expected for both groups as the study required scheduled visits and the use of comprehensive documentation. In contrast, several statistically significant effects in favor of the FDDA were observed for predefined and exploratory endpoints regarding aspects of targeting and managing the treatment of fatigue. Since many outcomes were studied beside the primary endpoint, multiple testing situation needs to be considered. Simple adjustment methods (like Bonferroni correction) are valid for uncorrelated outcomes. As the analysis showed, the outcomes are correlated to each other within specific factors (fatigue, satisfaction, treatment, others). Therefore, a formal adjustment was not foreseen. However, based on these 4 factors, one might use a lower alpha level of 0.0125 as a help to distinguish between more and less strong results. Finally, we did not analyze the cost-effectiveness of the FDDA. However, despite the aid being associated with more visits and investigations at baseline, more patients in this group reported satisfaction with their diagnosis. This may have positively influenced patient compliance with prescribed treatments, and ultimately the management of fatigue.

## Conclusions

This pilot study indicates that the FDDA, a structured diagnostic aid designed to assist primary care physicians in the differential diagnosis of unexplained fatigue, did not lead to a global improvement in patient-reported outcomes. However, it improved patients’ perception of their level of fatigue and satisfaction with care. Larger-scale studies are warranted to evaluate whether multidisciplinary aids like the FDDA can support PCPs in identifying the causes of unexplained fatigue in their patients.

## Supplementary Information

Supplementary Material 1.

Supplementary Material 2.

Supplementary Material 3.

## Data Availability

The raw data that support the findings of this study are a property of CSL Vifor. Restrictions apply to the availability of these data. However, they are available from the authors upon reasonable request and with permission from CSL Vifor.

## Abbreviations

CFS: Chronic Fatigue Syndrome
CGIC: Clinical Global Impression of Change
FAS: Full Analysis Set
FDDA: Fatigue Differential Diagnostic Aid
ICD: International Classification of Disease
PCP: Primary Care Physician
PGIC: Patient Global Impression of Change
PPS: Per Protocol Set
SAP: Statistical Analysis Plan
SFWG: Swiss Fatigue Working Group
