# Supplementary material for: Effects of a novel differential diagnosis aid for managing patients with unexplained fatigue in primary care: a prospective randomized, controlled, open and multicenter study in primary care
Source: BMC Prim Care. 2025 May 24;26:183. doi: 10.1186/s12875-025-02873-3 (PMC12102925; doi:10.1186/s12875-025-02873-3)
Supplement: Supplementary file 1 — Supplementary Material 1. [file 12875_2025_2873_MOESM1_ESM.pdf]

D D	M M	2 0 Y Y				
Day	Month	Year	Country	Doctor no.	Patient no.	ID

Questionnaire for fatigue assessment

The questionnaire should be filled out by the doctor.

A Questions on the duration of the tiredness/exhaustion, intensity and impact on quality of life

On a scale from 0 to 10:
On average, how severe/intense has your tiredness/exhaustion been during the last week?
(Tick what applies in line with the patient's statement)

0	1	2	3	4	5	6	7	8	9	10

0 = no tiredness/exhaustion, 10 = extreme tiredness/exhaustion

For how long have you felt this tiredness/exhaustion every day?
- ☐ <4 weeks
- ☐ 4–12 weeks
- ☐ >12 weeks – 6 months
- ☐ >6 months (indication of chronic fatigue syndrome)

On a scale from 0 to 10:
How much does tiredness/exhaustion impact on your private/professional/social life?
(Tick what applies in line with the patient's statement)

0	1	2	3	4	5	6	7	8	9	10

0 = no impact, 10 = severe impact

Have you already done something about this fatigue/tiredness?
- ☐ Yes → If so, what? ______________________
- ☐ No

B Questions regarding the self-assessment of the reasons/causes

What do you think are the reasons for your tiredness/exhaustion?
(Tick what applies in line with the patient's statement. Several answers are possible.)

☐ Lifestyle
(Poor/little sleep, internet addiction, medication abuse, alcohol, drugs, too much/too little exercise, long-distance travel, etc.)

☐ Psychological/affective cause
(Stress, depression, anxiety, burnout, etc.)

☐ Physical cause
(Infectious disease, metabolic problem, heart problem, etc.)

C Lifestyle questions

Do you take medication regularly?

☐ Yes → If yes, list medicines: ____________

☐ No

How many standard drinks of alcohol do you drink?

(1 standard drink = 1 glass of wine [1 dl] *or* 1 glass of beer [3 dl] or 1 glass of spirits [2 cl])[1]

☐ Number of standard drinks/week: ____________

☐ <1 standard drink/week or never: ____________

Do you use drugs (including cannabis or tranquilisers)?

☐ Yes → If yes, what and how often? ____________

☐ No

Do you fall asleep involuntarily during the day, for example when driving or in monotonous situations?

☐ Yes → If yes, in which situations? ____________

☐ No

Do you think you do not get enough hours of sleep?

☐ Yes → If yes, look for causes: ____________

☐ No

Do you suffer from one or more of the following sleep disorders?

Difficulty falling asleep	☐ Yes	☐ No
Trouble maintaining sleep	☐ Yes	☐ No
Waking early	☐ Yes	☐ No
Sleep that is not restful	☐ Yes	☐ No

Do the following events occur during your sleep?

☐ Snoring → If yes, e.g. STOP-Bang questionnaire

☐ Pause in breathing lasting several seconds (sleep apnoea)

Overall, do you exercise …[2]

☐ … moderately (e.g. walk for less than 2.5 hours/week)?

☐ … intensively (more than moderately or at least 1 hour of sport/day such as jogging, fitness exercises or another type of sport)?

☐ … not at all?

Have you lost or gained more than 5 percent body weight in the last 3 months?

☐ Yes → If yes, please describe the weight change (cause, extent, intentional):

____

☐ No

Are you vegetarian or vegan?

☐ Yes

☐ No

Have you previously missed work because of your tiredness/exhaustion?

☐ Yes

☐ No

1 Pasche S et al., Rev Med Suisse (2012);8:1831–1835. How to have a clear vision when considering all the different recommendations of moderate alcohol consumption?

2 Haskell WL et al., Med Sci Sports Exerc. (2007) Aug;39(8):1423–34. Physical activity and public health: updated recommendation for adults from the American College of Sports Medicine and the American Heart Association.

D Questions on mood, anxiety and secondary symptoms

Depression (PHQ-2)

**In the last two weeks, how often have you felt affected by the following complaints?** (Please tick the answer that best suits the patient)	Not at all	On some days	On more than half the days	Almost every day
1. Little interest or pleasure in your activities	0	1	2	3
2. Despondency, sadness or hopelessness	0	1	2	3
Subtotal	0 +	____ +	____ +	____
			**= Total score**	____

Total score ≥3 is indicative of depression → **PHQ-9 test**

Anxiety disorders (GAD-2)

**In the last two weeks, how often have you felt affected by the following complaints?** (Please tick the answer that best suits the patient)	Not at all	On some days	On more than half the days	Almost every day
1. Feelings of nervousness, anxiety or tension	0	1	2	3
2. Not being able to stop worrying or control worries	0	1	2	3
Subtotal	0 +	____ +	____ +	____
			**= Total score**	____

Total score ≥3 is indicative of an anxiety or panic disorder → **GAD-7 test**

Secondary symptoms

Do you have any other physical complaints?

☐ Yes → If yes, what are they? ____________________

☐ No

Stress

How often have you experienced stress in the past year because of…[3]

a) … problems at work?	☐ Not at all	☐ Rarely	☐ Now and then	☐ Often	☐ Very often
b) … problems at home?	☐ Not at all	☐ Rarely	☐ Now and then	☐ Often	☐ Very often
c) … financial problems?	☐ Not at all	☐ Rarely	☐ Now and then	☐ Often	☐ Very often

How would you rate your workload?

☐ Low

☐ Normal

☐ High

3 Rosengren A, Hawken S, Ounpuu S, et al. Lancet. 2004 Sep 11–17;364(9438):953–62. Association of psychosocial risk factors with risk of acute myocardial infarction in 11,119 cases and 13,648 controls from 52 countries (the INTERHEART study): case-control study.

E Further clarifications

Vital signs

Height ____________ cm   Weight ____________ kg   Pulse ____________ beats/min

Blood pressure (mm Hg) systolic ____________ diastolic ____________

For women: Do you have heavy periods (>7 days, of which >3 days heavy to very heavy)?

☐ Yes → If yes, how often do you change the tampon/pad? ____________ times/day
How long do your periods last? ____________ days

☐ No

For women: Could you be pregnant?

☐ Yes

☐ No

Men and women:
Have you noticed any unusual blood loss (in your urine, stool, nose bleeds, other)?

☐ Yes → If yes, what? ____________

☐ No

Have you donated blood in the last 12 months?

☐ Yes → If yes, how often? ____________

☐ No

Recommended laboratory analyses for assessment of fatigue

- CBC (recommendation: haemogram III, with three leucocyte subpopulations and lowering)
- Ferritin
- TSAT (transferrin saturation)
- CRP (if CRP not elevated, ferritin value probably reliable)
- TSH
- Glucose
- Electrolytes: Ca (albumin corrected), Na, K
- Creatinine
- Creatine kinase
- ALAT

Possible somatic causes of fatigue

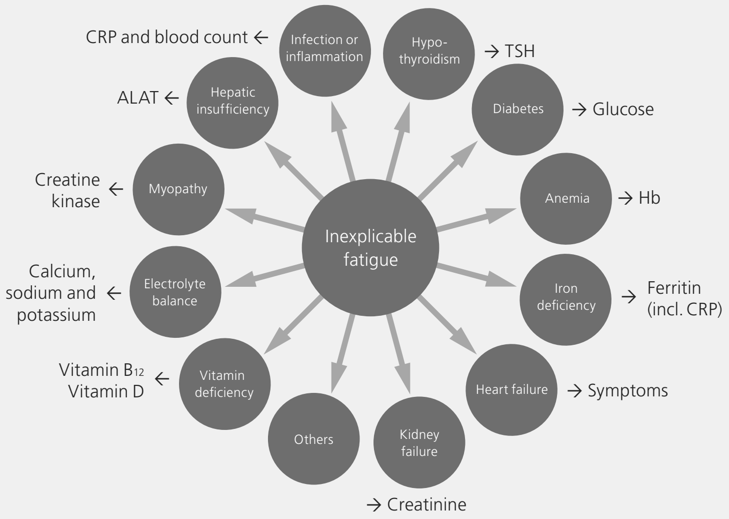

Further recommendations (according to previous findings)

- Vitamin B_{12}
- Vitamin D
- Folic acid

F Diagnostic

Lifestyle	Mental/affective disorder	Physical disorder
