# Supplementary material for: Effects of a novel differential diagnosis aid for managing patients with unexplained fatigue in primary care: a prospective randomized, controlled, open and multicenter study in primary care
Source: BMC Prim Care. 2025 May 24;26:183. doi: 10.1186/s12875-025-02873-3 (PMC12102925; doi:10.1186/s12875-025-02873-3)
Supplement: Supplementary file 2 — Supplementary Material 2. [file 12875_2025_2873_MOESM2_ESM.pdf]

Effects of a novel differential aid for managing patients with unexplained fatigue.
von Känel R, et al.

SUPPLEMENTARY MATERIAL

Supplementary Tables 1 to 3, and Supplementary Figures 1 to 3

Supplementary Table 1. Results for predefined endpoints.

Endpoint (No).	Endpoints	FDDA (n=40) vs usual care (n=53)	p-value
(1) PE	PGIC at 3 months, 7-point scale, *Mean (95%CI, range)*	2.7 (95%CI: 2.34-3.07, range: 1-6) vs. 2.6 (95%CI: 2.33-2.96, range: 1-5)	0.802
(2) SE	PGIC at 6 months, 7-point scale, *Mean (95%CI, range)*	2.6 (95%CI: 2.30-2.83, range: 1-4) vs. 2.5 (95%CI: 2.26-2.82, range: 1-5)	0.890
(3) SE	Patients experiencing a reduction in fatigue ≥1 point on a 1-10 scale, at 3 or 6 months,* *%*	97.4(37/38) vs. 78.4(40/51)	**0.011**
(4) SE	Time until an improvement of fatigue ≥1 point on a 1-10 NRS, *Median (Q1,Q3)*	14.0 (10,21) vs. 14.0 (7,21)	0.741
(5) SE	Mean number of points of fatigue reduction on a 1-10 NRS, *Mean (range)*	-2.0 (-6-1) vs. -2.1 (-7-1)	0.885
(6) SE	Patients with a PGIC response (=any improvement), *%*<br>at 3 months<br>at 6 months<br>at 3 or 6 months	 <br>78.4(29/37) vs. 78.6(33/43)<br>89.2(33/37) vs. 84.0(42/50)<br>92.1(35/38) vs. 94.1(48/51)	 <br>1.000<br>0.546<br>1.000
(7) SE	PCPs' confidence in the established diagnosis**, *Mean (range)*<br>Month 1<br>Month 3	 <br>0.7 (0-3) vs. 0.8 (0-3)<br>0.6 (0-2) vs. 0.6 (0-3)	 <br>0.609<br>0.750
(8) SE	PCPs' assessment of patient improvement (CGIC response), *Mean (range)*<br>Month 1<br>Month 3	 <br>3.1 (2-5) vs. 2.9 (1-4)<br>2.7 (1-6) vs. 2.4 (1-4)	 <br>0.318<br>0.231
(9a) SE	Patients satisfied or very satisfied with diagnosis ***, %<br>Month 1<br>Month 2<br>Month 3<br>Month 6	 <br>50.0(19/38) vs. 29.8(14/47)<br>50.0(19/38) vs. 40.0(20/50)<br>45.9(17/37) vs. 31.7(13/41)<br>64.9(24/37) vs. 52.0(26/50)	 <br>0.074<br>0.391<br>0.246<br>0.276

Endpoint (No).	Endpoints	FDDA (n=40) vs usual care (n=53)	p-value
(9b) SE	Patients satisfied or very satisfied with treatment ***, %<br>Month 1<br>Month 2<br>Month 3<br>Month 6	 <br>56.8(21/37) vs. 25.0(12/48)<br>44.7(17/38) vs. 36.0(18/50)<br>64.9(24/37) vs. 31.0(13/42)<br>59.5(22/37) vs. 48.0(24/50)	 <br>**0.004**<br>0.510<br>**0.003**<br>0.385
(10) SE	PCP-reported visits per patient for the same condition (fatigue), last available value, *Median (Q1,Q3)*	4.0 (3,4) vs. 3.0 (3,4)	**<0.001**
(11) SE	Referrals (Imaging (MRI, X-ray, etc.) or other health services	Free text data. Not analysable	
(12) SE	Time to final diagnosis**** [days], *Median (Q1,Q3)*<br>Month 1<br>Month 3	 <br>8.0(4,30) vs. 7.0(2,28)<br>14.0(7,30) vs. 7.0(2,30)	 <br>0.499<br>0.060

CGIC, Clinical global impression of change; EP, endpoint; NRS, numerical rating scale; PCP, primary care practitioner; PGIC, Patient global impression of change; P, primary endpoint; S, secondary endpoint.

* A patient experiencing a fatigue reduction ≥1 point at 3 or 6 months after the first visit, if at least at one visit was made (either at V4 or V5), was reported.

** The PCP expressed confidence in established diagnosis on a scale of 0 to 4: 0="valid"; 1="probably"; 2="possible"; 3="maybe".

*** Patient satisfaction was measured on a scale of 0 to 4: 0='Very unsatisfied'; 1='Unsatisfied'; 2='Neutral'; 3='Satisfied'; 4='Very satisfied'.

**** Physician-reported number of days at 1 and 3 months.

Supplementary Table 2. Overview of results obtained using simpler and more complex statistical methods for selected endpoints.

Endpoint variable	Visits	t-Test	Fisher's Exact Test [1)]	Model	Interaction term	`Group´-Effect (p-value)	`Visit´-Effect (p-value)	Interaction `Group*Visit´ (p-value)
**Primary endpoint 1 / Secondary endpoint 2: PGIC (FAS)**	V4	x				0.802	n.a.	n.a.
	V5 [3)]	x				0.890	n.a.	n.a.
	V2 to V5			x	x	0.5291	**0.0087**	0.3500
	V2 to V5			x		0.6612	**0.0123**	n.a.
	V2 & V4			x	x	0.9132	0.1362	0.8394
	V2 & V4			x		0.9476	0.1293	n.a.
**Secondary endpoint 5: Number of points of fatigue reduction (FAS)**	V2 to V5	x				0.885	n.a.	n.a.
	V2 to V5			x [2)]	x	0.9629	**0.0002**	0.4293
	V2 to V5			x [2)]		0.9450	**0.0003**	n.a.
	V2 & V4			x [2)]	x	0.7528	**0.0003**	0.6579
	V2 & V4			x [2)]		0.6377	**0.0002**	n.a.
**Secondary endpoint 7: GP confidence in the established diagnosis [excl. `no diagnosis´] (FAS)**	V2	x				0.609	n.a.	n.a.
	V4	x				0.750	n.a.	n.a.
	V2 & V4			x	x	0.7383	**0.0262**	0.7806
	V2 & V4			x		0.7760	**0.0225**	n.a.
**Secondary endpoint 8: CGIC (FAS)**	V2	x				0.318	n.a.	n.a.
	V4	x				0.231	n.a.	n.a.
	V2 & V4			x	x	0.2022	**0.0004**	0.8073
	V2 & V4			x		0.2105	**0.0004**	n.a.
**Secondary endpoint 9a: Patient satisfaction with diagnosis (FAS)**	V2		x			0.074	n.a.	n.a.
	V3 [3)]		x			0.391	n.a.	n.a.
	V4		x			0.246	n.a.	n.a.
	V5 [3)]		x			0.276	n.a.	n.a.
	V2 to V5			x	x	0.4964	**0.0488**	0.9257
	V2 to V5			x		0.4509	**0.0429**	n.a.
	V2 & V4			x	x	0.4515	0.3465	0.7216
	V2 & V4			x		0.3750	0.3548	n.a.
**Secondary endpoint 9b: Patient satisfaction with treatment (FAS)**	V2		x			**0.004**	n.a.	n.a.
	V3 [3)]		x			0.510	n.a.	n.a.
	V4		x			**0.003**	n.a.	n.a.
	V5 [3)]		x			0.385	n.a.	n.a.
	V2 to V5			x	x	0.2413	0.0552	0.1135
	V2 to V5			x		0.2426	**0.0386**	n.a.
	V2 & V4			x	x	**0.0292**	0.7863	0.4830
	V2 & V4			x		**0.0121**	0.8265	n.a.

[1)] Comparison of `very satisfied´ versus `other´ (which includes all remaining categories)
[2)] A baseline-adjusted model with repeated measurements (`Visit´), accounting for the clustered data structure (patients nested within physicians) and using `Group´ as a fixed effect was performed.
[3)] telephone consultation
n.a.: not applicable

This table provides an overview of the analyses conducted for the primary endpoint 1 / secondary endpoint 2, as well as secondary endpoints 5, 7, 8, 9a, and 9b.
For each **endpoint variable**, the corresponding analysis is indicated with an "x" for the respective Visits stated in the **Visits** column. The analyses performed include either:

- a **t-test**, or
- a **Fisher's Exact Test** (comparing `very satisfied´ versus `other´, which includes all remaining categories), or
- a **model** with repeated measurements (`Visit´), accounting for the clustered data structure (patients nested within physicians) and using `Group´ as a fixed effect:

- with or without the **interaction term** \`Group*Visit´, and
- incorporating all visits (i.e., including "face-to-face" consultations at Visits 2 and 4, as well as telephone consultations at Visits 3 and 5), or considering only Visits 2 and 4 (i.e., including only "face-to-face" consultations at Visit 2 and Visit 4).

The corresponding results (p-values) of the respective analyses are provided in the columns **\`Group´-Effect, \`Visit´-Effect** and **Interaction \`Group*Visit´**, if applicable (bold p-values indicate significant results). The interaction term \`Group*Visit´ is not significant in any of the models that contain the interaction term. The analysis of the secondary endpoint 9b using the repeated measures model shows a significant \`Group´-Effect (p: 0.0121) when Visit 2 and Visit 4 are considered, which was also demonstrated by Fisher's exact test. All statistical methods used to analyse the endpoints yielded the same results.

Supplementary Table 3. Primary care practitioner characteristics at baseline.

		**FDDA (n=15)**	**Usual care (n=22)**
Age (years) [at study entry]	Mean (Min, Max)	50.2 (34, 64)	48.7 (32, 62)
Level of experience (years)	Mean (Min, Max)	20.5 (2, 33)	18.3 (1, 36)
	Standard deviation	10.6	9.7
	Median (Q1, Q3)	26.0 (9, 29)	18.0 (11, 27)
Gender	Male, N (%)	9 (60.0%)	13 (59.1%)
	Female, N (%)	6 (40.0%)	9 (40.9%)

Supplementary Figure 1. Patient treatment patterns at 1 and 3 months.

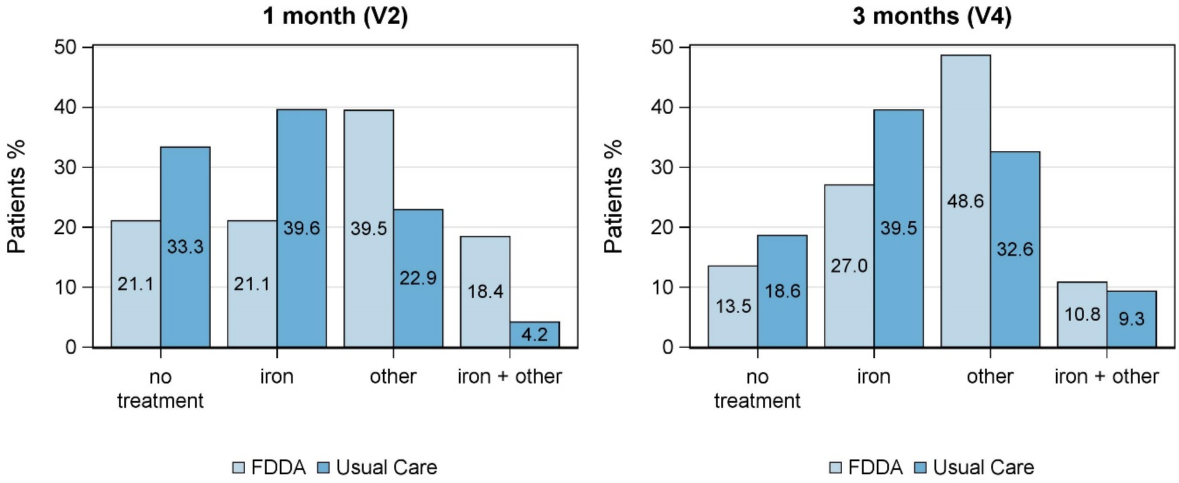

Patient treatments at 1 and 3 months. Light blue shading: FDDA. Dark blue shading: usual care.

Use of the Fatigue Differential Diagnostic Aid (FDDA) resulted in less patients receiving no treatment or iron treatment at 1 and 3 months, but receiving more other, non-iron replacement treatments, such as vitamins and micronutrients, complementary medicine, lipid lowering therapy, sleep-inducing agents, antithyroid treatment, antidepressants, asthma medication, or antibiotics.

Supplementary Figure 2. Treatments prescribed to patients against fatigue at 1 month.

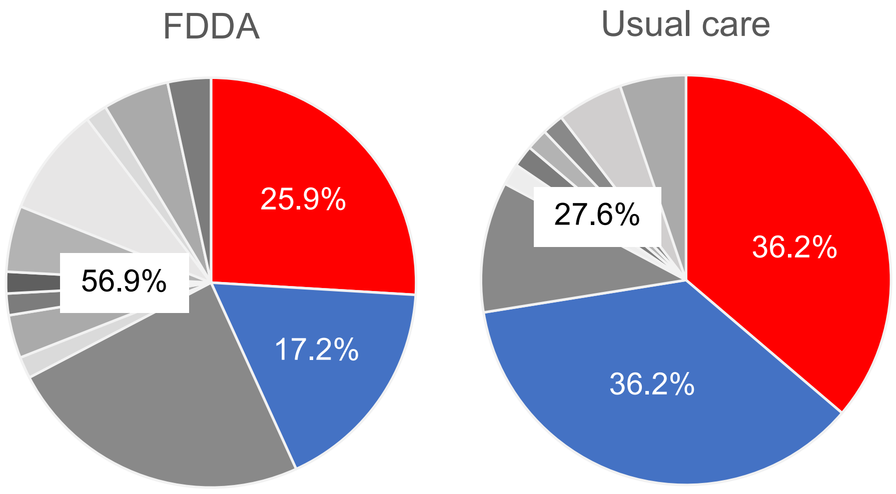

Blue: no treatment. Red: iron replacement therapy. Block shaded grey areas: non-iron replacement treatments, such as vitamins and micronutrients, complementary medicine, lipid lowering therapy, sleep-inducing agents, antithyroid treatment, antidepressants, asthma medication, or antibiotics.

Of note, a patient might contribute to several sectors.

Use of the Fatigue Differential Diagnostic Aid (FDDA) resulted in less patients receiving no treatment or iron treatment at 1 month, but more patients receiving other, non-iron replacement treatments.

Supplementary Figure 3. Treatments prescribed to patients against fatigue at 3 months.

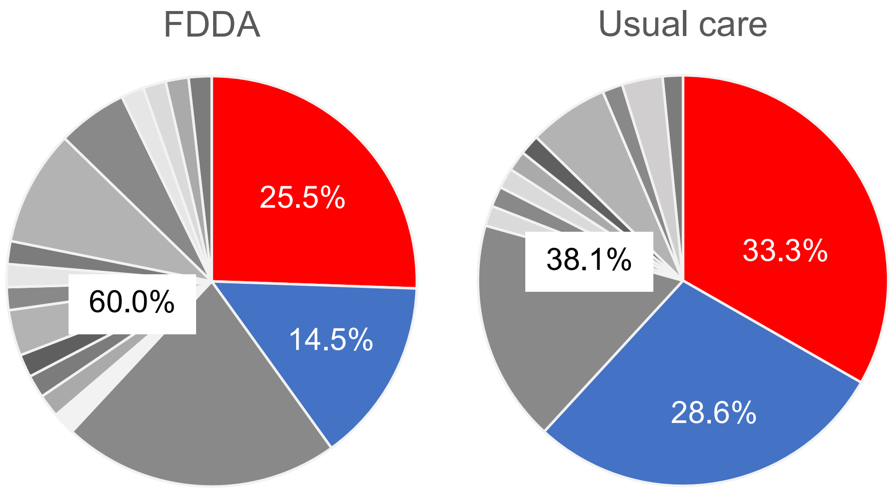

Blue: no treatment. Red: iron replacement therapy. Block shaded grey areas: non-iron replacement treatments, such as vitamins and micronutrients, complementary medicine, lipid lowering therapy, sleep-inducing agents, antithyroid treatment, antidepressants, asthma medication, or antibiotics.
Of note, a patient might contribute to several sectors.

Use of the Fatigue Differential Diagnostic Aid resulted in less patients receiving no treatment or iron treatment at 3 months, but more patients receiving other, non-iron replacement treatments.
